# Comparison of Four Formulas for Calculating LDL-Cholesterol with the Direct Homogeneous Method

**DOI:** 10.3390/biomedicines14061347

**Published:** 2026-06-15

**Authors:** Bosa Mirjanic-Azaric, Vera Lukić, Bojana Ivetic, Ana Sukur, Neda Milinković, Zana Radic Savic, Sanja Avram, Nataša Bogavac Stanojević, Ana Ninić

**Affiliations:** 1 Department of Medical Biochemistry, Faculty of Medicine, University of Banja Luka, 78000 Banja Luka, Bosnia and Herzegovina; anadobratic4@gmail.com (A.S.); zana.radic@med.unibl.org (Z.R.S.); 2 Institute of Laboratory Diagnostic, University Clinical Centre of the Republic of Srpska, 78000 Banja Luka, Bosnia and Herzegovina; bojana.ivetic@kc-bl.com (B.I.); sanja.avram@kc-bl.com (S.A.); 3 Department of Laboratory Diagnostics, Railway Healthcare Institute, 11000 Belgrade, Serbia; veralukic.lab@gmail.com; 4 Department for Medical Biochemistry, University of Belgrade—Faculty of Pharmacy, 11000 Belgrade, Serbia; nedan@pharmacy.bg.ac.rs (N.M.); natasa.bogavac@pharmacy.bg.ac.rs (N.B.S.); aninic@pharmacy.bg.ac.rs (A.N.)

**Keywords:** LDL-cholesterol, LDL-C estimation equations, Spasić–Kotur–Vujović formula, Friedewald formula, De Long formula, Martin–Hopkins formula

## Abstract

**Background/Objectives**: Low-density lipoprotein cholesterol (LDL-C) is a primary therapeutic target in cardiovascular risk assessment. While direct assays are available, LDL-C is frequently estimated using various formulas, whose performance can vary by population and lipid concentration. This study evaluates the analytical agreement and clinical classification performance of four formulas (Spasić–Kotur–Vujović, Friedewald, De Long, and Martin–Hopkins) against directly measured LDL-C. **Methods**: Lipid profiles from 3935 fasting adult patients were analyzed. LDL-C was directly measured via a homogeneous assay and compared with calculated values using linear regression and Bland–Altman analyses. Analytical bias and classification accuracy were assessed across stratified lipid levels. **Results**: Among the evaluated equations, the Spasić–Kotur–Vujović formula showed the closest agreement with directly measured LDL-C. It had the lowest mean absolute bias and mean percentage differences across most lipid strata, as well as narrower limits of agreement compared with Friedewald, De Long, and Martin–Hopkins formulas. Although all formulas showed strong correlations with directly measured LDL-C (Pearson’s r correlation coefficient = 0.956–0.958; intraclass correlation coefficient > 0.974), systematic underestimation of LDL-C was observed for all equations and became more pronounced with increasing total cholesterol, LDL-C, triglycerides, and non-high-density lipoprotein cholesterol concentrations. The Spasić–Kotur–Vujović formula achieved the highest overall classification accuracy (70%), followed by De Long (68%), Martin–Hopkins (61%), and Friedewald (58%). **Conclusions**: Despite strong correlations, clinically relevant differences in bias and classification exist among LDL-C formulas. The Spasić–Kotur–Vujović formula demonstrated superior agreement and accuracy in this population, suggesting it is a more reliable tool for routine laboratory practice. It should be noted that these findings reflect agreement with the specific homogeneous LDL-C assay used on the Alinity c platform (Abbott Laboratories,, Chicago, IL, USA) and may not be directly generalizable to other analytical systems.

## 1. Introduction

Low-density lipoprotein cholesterol (LDL-C) is a key marker in cardiovascular disease (CVD) risk assessment and the primary target for lipid-lowering therapy in current clinical guidelines [1]. LDL-C is also the main therapeutic target in both primary and secondary prevention strategies, including statin and non-statin lipid-lowering therapies [2]. Reliable LDL-C measurement is necessary for clinical decision-making and for monitoring the response to lipid-lowering therapy in routine practice.

Ultracentrifugation-based beta-quantification is widely regarded as the reference method for LDL-C determination. Due to its technical complexity and resource requirements, this method is not routinely used in standard clinical laboratory workflows. Direct homogeneous LDL-C assays have therefore been developed for use on automated analyzers. However, these assays are associated with higher costs, platform-dependent analytical variability, and limited availability in some laboratory settings, particularly in low- and middle-resource environments [3]. As a result, calculated LDL-C values remain the most widely used method for LDL-C assessment in routine clinical practice worldwide.

Among calculation-based methods, the Friedewald formula is still the most commonly used equation. This formula estimates LDL-C using total cholesterol (TC), high-density lipoprotein cholesterol (HDL-C), and triglycerides (TG), assuming a fixed relationship of 2.2 between TG and very low-density lipoprotein cholesterol (VLDL-C) when expressed in mmol/L [4]. Although simple and cost-effective, the Friedewald formula has well-recognized limitations. Its accuracy declines substantially at higher TG concentrations, it is unreliable in non-fasting samples, and it is not recommended for patients with certain dyslipidemias, including familial dysbetalipoproteinemia [5].

To address these limitations, many alternative LDL-C estimation formulas have been developed [6]. Among the most extensively studied are the De Long, Martin–Hopkins and Spasić–Kotur–Vujović equations [7,8,9]. The De Long formula represents a modification of the Friedewald approach but retains a fixed TG-to-VLDL-C ratio and therefore remains susceptible to TG-related inaccuracies [7]. The Martin–Hopkins formula incorporates an adaptive TG-to-VLDL-C factor derived from a stratified lookup table based on TG and non-HDL-C concentrations, allowing for a more individualized LDL-C estimation and improved performance in certain populations, particularly at borderline or moderately elevated TG levels [8]. The LDL-C estimation formula developed in Serbia (the Spasić–Kotur–Vujović formula) incorporates a fixed TG-to-VLDL ratio, which is higher than in the Friedewald and De Long formulas [9]. Compared to the Friedewald formula, the Spasić–Kotur–Vujović formula improved correct classification according to The National Cholesterol Education Program (NCEP) Adult Treatment Panel III (ATP III) LDL-C risk categories, generally minimizing underestimation and reducing misclassification near the therapeutic decision threshold, especially at 4.14 mmol/L. However, it is sensitive to TG-related inaccuracies [9].

Previous studies have shown that the analytical performance of LDL-C estimation formulas varies significantly across TG concentrations, LDL-C ranges, and lipid phenotypes. Systematic underestimation of LDL-C by calculation-based methods has been repeatedly reported, particularly at higher TG and LDL-C concentrations, raising concerns about clinically relevant misclassification of patients [5,10,11]. This is clinically important because current treatment algorithms rely on guideline-defined LDL-C categories, and even modest analytical deviations near decision thresholds may affect patient classification and subsequent therapeutic management [1,2]. The clinical importance of formula selection is further illustrated by a recent study demonstrating that, in hospitalized patients with diabetes, choice of the LDL-C estimation equation significantly affected patient classification into guideline-recommended LDL-C targets [12].

Lipid distributions vary between populations due to dietary, metabolic, and lifestyle factors. Therefore, LDL-C estimation formulas developed and validated in one population may not perform optimally in other clinical settings. Evidence from regional validation studies indicates that locally derived or validated equations may provide improved analytical agreement in populations with similar metabolic characteristics [10,11]. However, systematic comparative evaluations across clinically relevant lipid strata, incorporating both analytical agreement and classification accuracy, remain limited.

This study compares the analytical performance of four LDL-C estimation formulas (Spasić–Kotur–Vujović, Friedewald, De Long, and Martin–Hopkins) with directly measured LDL-C (D-LDL-C) in a routine clinical laboratory setting. Specifically, our aims are to assess agreement between calculated and D-LDL-C values, evaluate classification accuracy across treatment-relevant LDL-C categories, and examine systematic bias and limits of agreement across stratified ranges of LDL-C, TC, TG, and non-HDL-C.

## 2. Materials and Methods

### 2.1. Study Design and Population

This cross-sectional study was conducted using data of 3935 patients retrieved from the laboratory information system (LIS) of the University Clinical Center of the Republic of Srpska in Banja Luka, Bosnia and Herzegovina. Adult patient samples obtained during routine lipid profile testing between January 2022 and February 2024 were included. Samples were obtained after an overnight fast.

Eligible samples were required to have complete measurements of TC, HDL-C, TG, and D-LDL-C, all obtained from the same blood sample. Samples were included in the analysis only if TG concentrations fell within the applicability range of the respective LDL-C estimation formula. Formula-specific exclusions were applied according to published applicability thresholds. For the Friedewald, De Long, and Spasić–Kotur–Vujović formulas, samples with TG ≥ 4.5 mmol/L were excluded as specified in the original publications. For the Martin–Hopkins formula, although the lookup table extends to TG < 9.04 mmol/L, the same upper TG threshold of 4.5 mmol/L was applied to maintain methodological consistency across all four formulas.

All laboratory data were fully anonymized prior to analysis. The study protocol was approved by the Ethics Committee of the University Clinical Center of the Republic of Srpska (approval No. 01-19-131-2/24, issued on 20 March 2024).

### 2.2. Laboratory Measurements

Blood samples were collected and processed according to standard laboratory procedures. Serum lipid parameters, including TC, HDL-C, and TG, were measured using standardized enzymatic assays on the Alinity c clinical chemistry analyzer (Abbott Laboratories, Chicago, IL, USA), in accordance with the manufacturer’s instructions.

D-LDL-C was determined using a homogeneous D-LDL-C assay on the same analytical platform. Although not a reference method in the strict analytical sense, D-LDL-C served as the comparative reference for evaluating the analytical performance of calculated LDL-C estimation formulas, reflecting routine clinical laboratory practice.

Reproducibility of the D-LDL-C assay was monitored through daily internal quality control. The observed analytical imprecision remained within the manufacturer’s declared within-laboratory precision specifications (CV ≤ 1.6%) throughout the study period. The laboratory also participated in an external quality assessment program.

### 2.3. Calculated Lipid Parameters

Non-HDL-C was calculated as the difference between TC and HDL-C and was included in stratified analyses as an additional clinically relevant lipid parameter.

LDL-C concentrations were calculated using four different estimation formulas [4,7,8,9].
Formula nameEquationFriedewaldF-LDL-C (mmol/L) = TC − HDL-C − TG/2.2De LongDL-LDL-C (mmol/L) = TC − HDL-C − TG/2.7Martin–HopkinsMH-LDL-C (mmol/L) = TC − HDL-C − TG/adjustable factorSpasić–Kotur–VujovićSKV-LDL-C (mmol/L) = TC − HDL-C − TG/3

The adjustable factor in the Martin–Hopkins formula was directly calculated for each examinee when TC, HDL-C, and TG concentrations in mg/dL were entered in the Excel worksheet table available online [8].

All four formulas for LDL-C estimation were applied using identical input parameters obtained from the same blood sample, allowing direct comparison between methods. They were selected to represent similar methodological approaches: the Friedewald formula as the conventional equation most widely used in routine practice; the Spasić–Kotur–Vujović formula as a locally developed, population-validated model with a fixed ratio for VLDL-C; and the De Long formula as a representative fixed-ratio modification of the Friedewald approach, similar to but distinct from the Spasić–Kotur–Vujović formula. These formulas were chosen because they have increasing TG-to-VLDL-C ratios of 2.2 (Friedewald), 2.7 (De Long), and 3 (Spasić–Kotur–Vujović), in order to determine the influence of this ratio on the calculated LDL-C concentration. The adaptive, lookup-table-based method Martin–Hopkins formula was chosen as a widely recommended and validated adaptive method incorporating variable TG-to-VLDL-C ratios.

### 2.4. Data Stratification

Stratified analyses were conducted to assess correct classification and potential misclassification (under- and overestimation) of calculated LDL-C values when D-LDL-C was considered the true value across different lipid concentration ranges. Stratification thresholds were chosen based on lipid concentration ranges commonly used in cardiovascular risk assessment and guideline-based treatment decisions [13]. LDL-C values were stratified in the following categories: ≤2.5, 2.6–3.3, 3.4–4.1, 4.2–4.8, and ≥4.9 mmol/L. Comparisons between the methods were also performed when samples were stratified according to recommended TC levels (≤4.1, 4.2–5.1, 5.2–6.1, 6.2–7.1, and ≥7.2 mmol/L), TG levels (≤1.1, 1.2–1.6, 1.7–2.2, 2.3–2.8, and 2.9–4.4 mmol/L), and non-HDL-C levels (≤2.5, 2.6–3.2, 3.3–3.8, 3.9–4.5, and ≥4.6 mmol/L).

### 2.5. Statistical Analysis

Statistical analyses were performed using Microsoft Excel Worksheet (Washington, DC, USA) and MedCalc Software v. 12.5.0.0 (Ostend, Belgium). Student’s *t*-test for paired samples was used to compare means. Data are given as mean ± standard deviation (SD). The statistical significance was set at *p* < 0.05. The associations between D-LDL-C values and LDL-C calculated by different formulas were assessed using linear regression analysis. Data were presented as Pearson’s r correlation coefficient and R^2^ coefficient of determination. A Pearson’s r coefficient of 0.9 or higher indicated a very strong correlation; a value from 0.7 to 0.9 indicated a strong correlation between variables; a value from 0.4 to 0.7 indicated a moderate correlation between variables [14]. The intraclass correlation coefficient (ICC) was used to evaluate the agreement or reliability between D-LDL-C values and LDL-C values obtained by different formulas. An ICC above 0.90 indicates excellent reliability [15]. Data are presented as ICC and 95% confidence interval (CI).The concordance correlation coefficient (CCC), which includes precision (Pearson’s r correlation coefficient) and accuracy (bias-corrected factor, Cb), was used to determine the degree to which pairs of observations fall on the 45° line through the origin. Data were reported as CCC, 95% CI, r, and Cb. Cb values between 0.95 and 0.99 indicate substantial agreement, and values above 0.99 indicate almost perfect agreement [16].

Absolute bias and limits of agreement (LoA), defined as LoA − (−1.96 × SD) and LoA + (+1.96 × SD), were determined by Bland–Altman analysis. The mean percentage difference (%ΔLDL-C) was also calculated using the following formula: %ΔLDL-C = [(D-LDL-C) − (calculated LDL-C)]/(D-LDL-C) × 100.

## 3. Results

In Table 1, mean LDL-C concentrations are presented and compared with the recommended values for LDL-C, TC, TG, non-HDL-C, and for the overall dataset. Significant differences were observed between all calculated LDL-C values and those obtained by direct measurement. All formulas, across all stratification levels of D-LDL-C, TC, TG, and non-HDL-C, produced values significantly lower than D-LDL-C, except for SKV-LDL-C when D-LDL-C ≤ 2.5 mmol/L. Across the entire dataset of 3935 examinees, LDL-C values obtained by all four formulas showed a very strong positive linear correlation (precision) with D-LDL-C, as demonstrated by Pearson’s r correlation coefficients of 0.958, 0.956, 0.958, and 0.957 for the Spasić–Kotur–Vujović, Friedewald, De Long, and Martin–Hopkins formulas, respectively (Table 1).

**Figure 1:**
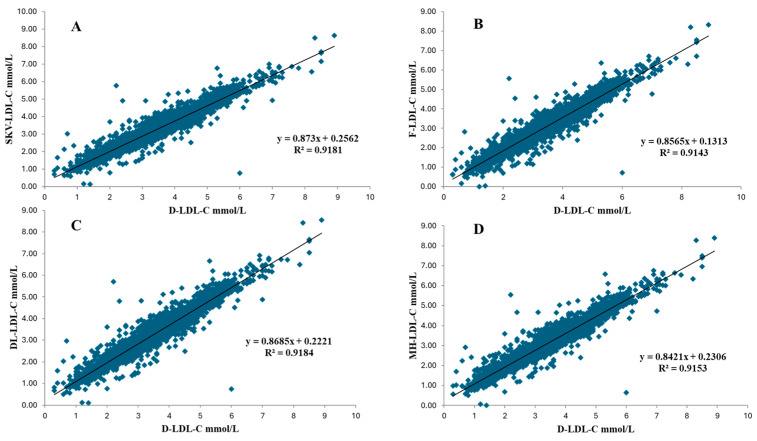
Linear regression analysis of LDL-C values calculated using the (**A**) Spasić–Kotur–Vujović, (**B**) Friedewald, (**C**) De Long, and (**D**) Martin–Hopkins formulas versus directly measured LDL-C (D-LDL-C) concentrations in the overall study population (*n* = 3935). The figure shows the regression equations and coefficients of determination (R^2^) for each formula. Intercepts and slopes were tested against the ideal values of 0 and 1, respectively, and all formulas showed significant deviations, indicating both systematic and proportional errors. High coefficients of determination (R^2^ > 0.90) demonstrate a very strong linear relationship between calculated LDL-C and D-LDL-C values, with more than 91% of the variability in calculated LDL-C explained by D-LDL-C concentrations.

The regression equations for the linear function are presented in Figure 1A for the Spasić–Kotur–Vujović formula, Figure 1B for the Friedewald formula, Figure 1C for the De Long formula, and Figure 1D for the Martin–Hopkins formula. Intercepts and slopes for all four formulas were significantly different from 0 and 1 (*p* < 0.001 and *p* < 0.001, respectively), indicating significant systematic and proportional errors. Furthermore, the correlation between the tested and direct homogeneous methods across the whole dataset was confirmed, as R^2^ > 0.90 (Figure 1) indicated a strong relationship between each formula and the direct homogeneous method. More than 91% of the variation in calculated LDL-C values using all four formulas could be explained by D-LDL-C.

The ICC between the SKV-LDL-C, F-LDL-C, DL-LDL-C, MH-LDL-C and D-LDL-C were ICC = 0.976, 95% CI: 0.975–0.979; ICC = 0.975, 95% CI: 0.973–0.979; ICC = 0.976, 95% CI: 0.975–0.979 and ICC = 0.974; 95% CI: 0.972–0.975, respectively, for the overall dataset.

The CCC, 95% CI for CCC, and accuracy values for the Spasić–Kotur–Vujović formula were 0.944 (0.941–0.947), 0.985; for the Friedewald formula, 0.907 (0.902–0.912), 0.949; for the De Long formula, 0.937 (0.933–0.940), 0.977; and for the Martin–Hopkins formula, 0.917 (0.912–0.921), 0.958, for the overall dataset.

### 3.1. Agreement Between Calculated and Directly Measured LDL-C Across Lipid Strata

#### 3.1.1. Stratification by D-LDL-C

When participants were stratified by D-LDL-C concentration, increasing differences (mean absolute bias and mean percentage bias) between calculated LDL-C and D-LDL-C were observed as LDL-C levels increased (Table 2). In the lowest LDL-C category (≤ 2.5 mmol/L), SKV-LDL-C and DL-LDL-C had mean values closest to D-LDL-C; the Spasić–Kotur–Vujović formula overestimated D-LDL-C by 0.02 mmol/L, while the De Long formula underestimated D-LDL-C by the same amount (Table 2). At higher LDL-C concentrations (≥ 2.6 mmol/L) across different recommended LDL-C values, all formulas underestimated D-LDL-C, with the smallest mean absolute bias and mean percentage difference for the Spasić–Kotur–Vujović formula. The highest mean absolute bias and mean percentage difference were observed for the Friedewald formula, followed by the Martin–Hopkins formula. For D-LDL-C values ≤ 2.5 mmol/L and ≥ 4.9 mmol/L, Pearson’s r for all formulas ranking from 0.7 to 0.9 indicated strong correlation between variables. For D-LDL-C values ≥ 2.6 mmol/L and ≤ 4.8 mmol/L, Pearson’s r for all formulas ranking from 0.4 to 0.7 indicated a moderate correlation between variables.

#### 3.1.2. Stratification by TC

In all TC categories, the mean LDL-C values estimated by all four formulas were significantly lower than D-LDL-C (Table 1). SKV-LDL-C had the closest values to D-LDL-C. The mean absolute differences between calculated values and D-LDL-C increased with higher TC concentrations (Table 2). The smallest differences were observed for the Spasić–Kotur–Vujović formula in each TC category compared to the other formulas. In contrast, the highest differences were found with the Friedewald formula followed by the Martin–Hopkins formula. As TC increased, the magnitude of underestimation increased for all equations, reaching its peak at concentrations above the recommended value of 7.2 mmol/L. A similar pattern was observed for the mean percentage difference. The lowest mean percentage difference was evident for the Spasić–Kotur–Vujović formula across all TC recommended values compared to the other formulas. The highest mean percentage difference (4.64%) between D-LDL-C and SKV-LDL-C was in the 5.2–6.1 mmol/L TC category. However, when comparing D-LDL-C with F-LDL-C, DL-LDL-C, and MH-LDL-C, the highest mean percentage differences (10.11%, 6.06%, and 8.38%, respectively) were observed at lower TC concentrations, ranging 4.2–5.1 mmol/L (Table 2). For all formulas, a Pearson’s r from 0.7 to 0.9 indicated a strong correlation between calculated and D-LDL-C in all TC-recommended values except for TC between 6.2 and 7.1 mmol/L (Table 1).

#### 3.1.3. Stratification by TG

In all TG categories, the mean LDL-C values estimated by all four formulas were significantly lower than D-LDL-C (Table 1). SKV-LDL-C was the closest to D-LDL-C. The mean absolute bias between calculated LDL-C and D-LDL-C increased up to TG ≤ 2.2 mmol/L for all formulas except for Friedewald, where they increased up to 4.4 mmol/L TG (Table 2). The same was observed for the mean percentage difference for the Friedewald formula. The lowest mean percentage difference was observed for the Spasić–Kotur–Vujović formula across all TG categories compared to the other formulas. However, the highest mean percentage difference (4.03%) between D-LDL-C and SKV-LDL-C was in the 1.2–1.6 mmol/L TG category. When comparing D-LDL-C with DL-LDL-C and MH-LDL-C, the highest mean percentage differences, 5.58% and 9.18%, were in the 1.2–1.6 mmol/L and ≤1.1 mmol/L TG categories, respectively (Table 2). A Pearson’s r > 0.9 in all TG categories suggested a very strong linear relationship between calculated D-LDL-C values (Table 1).

#### 3.1.4. Stratification by Non-HDL-C

In all non-HDL-C categories, the mean LDL-C values estimated by all four formulas were significantly lower than D-LDL-C (Table 1). SKV-LDL-C was the closest to D-LDL-C. With increasing non-HDL-C levels, calculated LDL-C values increasingly underestimated D-LDL-C (Table 2). The smallest differences were observed for the Spasić–Kotur–Vujović formula in each non-HDL-C category compared to the other formulas. In contrast, the highest differences were found with the Friedewald formula followed by the Martin–Hopkins formula. The lowest mean percentage difference was observed for the Spasić–Kotur–Vujović formula across all non-HDL-C categories compared to the other formulas. The highest mean percentage differences (5.16%, 10.39%, 6.59% and 8.57%) between D-LDL-C and SKV-LDL-C, F-LDL-C, DL-LDL-C, and MH-LDL-C, respectively, were in the 3.9–4.5 mmol/L non-HDL-C category (Table 2). For all formulas, linearity and precision (Pearson’s r from 0.7 to 0.9) were strong for all equations and D-LDL-C in all non-HDL-C categories except for non-HDL-C ≤ 2.5 mmol/L (Table 1).

Stratified analysis revealed a clear pattern of increasing bias and widening limits of agreement at higher lipid concentrations (Table 2). This deterioration in agreement was most evident for F-LDL-C and MH-LDL-C, particularly in strata with elevated TG and non-HDL-C. In contrast, SKV-LDL-C and DL-LDL-C maintained comparatively lower bias and narrower limits of agreement across lipid categories.

### 3.2. Classification Accuracy Across Treatment-Relevant LDL-C and Other Lipid Categories

The ability of the four formulas to correctly classify participants into guideline-defined LDL-C treatment categories varied substantially (Table 3).

Overall classification accuracy was highest for the Spasić–Kotur–Vujović formula (70%), followed by De Long (68%), Martin–Hopkins (61%), and Friedewald (58%). On the other hand, the percentage of underestimated values was the smallest for SKV-LDL-C (27%), followed by DL-LDL-C (29%), MH-LDL-C (37%), and F-LDL-C (40%). For all formulas, classification accuracy decreased markedly from low to intermediate and moderately elevated LDL-C categories, reaching its lowest levels in the 4.2–4.8 mmol/L range, while partial recovery of accuracy was observed in the highest LDL-C category (≥4.9 mmol/L). In the LDL-C range of 4.2–4.8 mmol/L, correct classification was achieved in 39%, 35%, 23% and 20% of cases using the Spasić–Kotur–Vujović formula, De Long formula, Martin–Hopkins formula and Friedewald formula, respectively. Only for LDL-C values ≤ 2.5 mmol/L the Friedewald formula demonstrated the highest percentage of correct classification (98%), compared to other formulas.

Across TC strata, broadly similar patterns were observed for all formulas, with reduced classification performance in intermediate to high lipid ranges and improved performance in the lowest and highest strata. In non-HDL-C categories, classification performance declined from the lowest to highest categories for all formulas. The highest percentage of correctly classified and the lowest percentage of underestimated data were evident for the Spasić–Kotur–Vujović formula in all TC and non-HDL-C classification values, except for non-HDL-C ≤ 2.5 mmol/L, where all formulas demonstrated the same highly correct classification (97%) and low underestimation rate (3%). The highest percentage of correct patient classification (95%) was achieved by the Spasić–Kotur–Vujović formula for TC levels above 7.2 mmol/L.

Across TG strata, classification accuracy declined from low to high TG concentrations for all formulas. However, the Spasić–Kotur–Vujović formula showed the highest percentage of correctly classified data and the lowest percentage of underestimated data in all TG ranges. The Friedewald formula, followed by the Martin–Hopkins formula, consistently showed the lowest classification accuracy across lipid subgroups. In contrast, the De Long formula performed very similarly to the Spasić–Kotur–Vujović formula.

## 4. Discussion

### 4.1. Principal Findings in the Analytical and Clinical Context

In this large outpatient cohort, we compared four LDL-C estimation formulas (Spasić–Kotur–Vujović, Friedewald, De Long, and Martin–Hopkins) against directly measured LDL-C from a homogeneous assay, with stratification across clinically relevant lipid values. The analysis revealed several findings relevant to both analytical performance and clinical interpretation.

First, although the Friedewald, De Long, and Spasić–Kotur–Vujović formulas do not account for heterogeneity in TG-to-VLDL-C ratios, the last formula correctly classified more patients and underestimated fewer within the recommended lipid levels than any other formula (Table 3). Overall misclassification rates differed from those reported in other populations, highlighting the population-dependent performance of LDL-C equations [11]. Nevertheless, the Spasić–Kotur–Vujović formula consistently showed the lowest misclassification rates across LDL-C, TC, TG, and non-HDL-C categories. All calculation-based formulas showed systematic underestimation of D-LDL-C, and this underestimation became more pronounced with increasing TC, LDL-C, TG, and non-HDL-C. Similar limitations of LDL-C estimation formulas, particularly in dyslipidemic states and at higher TG concentrations, have been reported both in studies evaluating widely used equations such as Friedewald and Martin–Hopkins [5,10] and in larger comparative analyses including multiple LDL-C formulas [6,11,17].

Second, the magnitude of underestimation and the degree of analytical agreement differed meaningfully among formulas. Across lipid strata, the Spasić–Kotur–Vujović formula demonstrated the smallest mean bias, the narrowest limits of agreement, and the highest classification accuracy, whereas the Friedewald formula showed the largest deviations and the poorest categorical performance. Third, misclassification into guideline-defined LDL-C categories increased substantially at higher LDL-C concentrations for all formulas, indicating that even modest absolute analytical differences translate into clinically relevant category shifts when LDL-C values lie near therapeutic decision thresholds [1,2,6,10].

### 4.2. Analytical Agreement and Bias Beyond Correlation Metrics

Despite high Pearson’s r (>0.950), R^2^ (>0.914), CCC (>0.900), and ICC (>0.974) for all formulas, these metrics alone were insufficient to establish clinical interchangeability because all equations exhibited systematic and proportional errors in linear regression analyses.

Despite maintaining high overall correlations (r = 0.956–0.958), the Friedewald and Martin–Hopkins formulas reached mean absolute biases of 0.53 and 0.49 mmol/L (Table 2) in the clinically critical 4.2–4.8 mmol/L LDL-C range, sufficient to shift patients across guide-line-defined thresholds. This translated into correct classification in only 20% and 23% of cases in that interval, respectively (Table 3)—a striking illustration that high correlation coefficients do not ensure clinical interchangeability.

In contrast, the Spasić–Kotur–Vujović formula demonstrated not only high correlation but also consistently smaller bias and narrower limits of agreement across LDL-C, TC, TG, and non-HDL-C strata. Similarly, Khan et al. showed in a cohort of 1075 patients that among nine examined LDL-C equations, the Spasić–Kotur–Vujović formula exhibited the lowest bias and high correlation with D-LDL-C [18]. Analytically, this indicates closer alignment with the D-LDL-C assay across a broad range of lipid phenotypes and suggests a lower probability of clinically meaningful misclassification in routine practice [9,19]. This observation is further supported by the recent systematic review and meta-analysis by Ephraim et al., which demonstrated that the Spasić–Kotur–Vujović LDL-C equation exhibits a stronger correlation with D-LDL-C (r = 0.95) compared with the Friedewald formula (r = 0.88), reinforcing its analytical robustness across diverse populations [20].

### 4.3. TG as the Dominant Modifier of Formula Performance

TG concentration emerged as the most influential determinant of formula performance. At low TG levels (≤1.1 mmol/L), mean absolute biases ranged from 0.16 mmol/L (Spasić–Kotur–Vujović) to 0.30 mmol/L (Martin–Hopkins) (Table 2), consistent with acceptable performance under normotriglyceridemic conditions [5,6,10]. With rising TG, divergence between formulas became pronounced.

The Friedewald formula showed the most pronounced increase in bias with rising TG concentrations, with mean absolute bias increasing from 0.26 mmol/L at TG ≤ 1.1 mmol/L to 0.51 mmol/L at TG 2.9–4.4 mmol/L. In contrast, the other formulas demonstrated only modest and non-directional variations across TG strata: the Spasić–Kotur–Vujović formula ranged from 0.10 to 0.19 mmol/L, the De Long formula from 0.18 to 0.25 mmol/L, and the Martin–Hopkins formula from 0.30 to 0.13 mmol/L (Table 2). Across TG categories, the Spasić–Kotur–Vujović formula consistently demonstrated the lowest mean absolute bias and the smallest mean percentage differences (0.15–4.03%). This translated into the highest or near-highest correct classification rates (75%, 69%, 69%, 68%, and 64% across increasing TG strata) and the lowest underestimation rates (24–29%) compared with the other formulas (Table 3).

The Friedewald formula relies on a fixed TG-to-VLDL-C ratio, an assumption known to break down in metabolic conditions characterized by altered lipoprotein composition, such as insulin resistance, obesity, type 2 diabetes, and mixed dyslipidemia [4,5]. The Martin–Hopkins formula was developed to address this limitation by incorporating an adaptive TG-to-VLDL-C factor derived from large datasets [8,21]. While this approach has been shown to improve LDL-C estimation in some populations, its performance depends on how closely the lipid distributions of the target population resemble those of the derivation cohorts [10,11,17,22,23]. In the present study, the Martin–Hopkins formula did not outperform the Spasić–Kotur–Vujović or De Long formulas, suggesting that population-specific differences in lipoprotein composition may limit the generalizability of adaptive lookup-table approaches. The Martin–Hopkins adjustable factor was derived from a different population structure and may therefore be less applicable to the lipid distribution observed in the present cohort.

Like the Friedewald and De Long formulas, the Spasić–Kotur–Vujović formula is based on a fixed TG-to-VLDL ratio. Validated in a population with similar regional, dietary, and metabolic characteristics, it appears to provide a TG correction factor that better reflects the lipid profile of this cohort compared to Friedewald and De Long.

### 4.4. Performance Across Non-HDL-C Strata

Stratification by non-HDL-C provided further insight into formula performance in patients with more complex and atherogenic lipid profiles. Non-HDL-C reflects the total cholesterol burden carried by all atherogenic lipoproteins, including LDL, VLDL, and remnant particles, and is increasingly highlighted in clinical guidelines [1,2].

As non-HDL-C increased from ≤2.5 mmol/L to 3.9–4.5 mmol/L, mean absolute bias increased from 0.05 to 0.22 mmol/L for the Spasić–Kotur–Vujović formula, from 0.18 to 0.42 mmol/L for Friedewald, from 0.08 to 0.28 mmol/L for De Long, and from 0.14 to 0.36 mmol/L for Martin–Hopkins (Table 2). The largest deviations were consistently observed for the Friedewald and Martin–Hopkins formulas. These findings are consistent with previous reports indicating that LDL-C estimation becomes less reliable in the presence of elevated remnant lipoproteins and heterogeneous LDL particle composition [5,6,17,24]. Notably, patients with elevated non-HDL-C are often at highest CVD risk and are the most likely to require intensive lipid-lowering therapy. Underestimation of LDL-C in this context may therefore contribute to undertreatment and delayed achievement of guideline-recommended targets.

The comparatively smaller mean absolute bias observed with the Spasić–Kotur–Vujović formula (Table 2) in high non-HDL-C strata suggests it may be particularly suitable for patients with mixed dyslipidemia and elevated atherogenic burden.

### 4.5. Clinical Implications of LDL-C Misclassification

The analytical differences observed among formulas translated directly into clinically relevant differences in classification accuracy. Across all lipid strata, the Spasić–Kotur–Vujović formula achieved the highest proportion of correct classification into guideline-defined LDL-C categories, while the Friedewald formula consistently showed the lowest accuracy, followed by Martin–Hopkins and De Long formulas (Table 3). The Spasić–Kotur–Vujović formula was in substantial agreement with D-LDL-C, showing a smaller systematic error at clinically relevant recommended LDL-C values of 2.5 mmol/L, 3.4 mmol/L, and 4.2 mmol/L.

The decline in classification accuracy at higher LDL-C concentrations is especially concerning, as these ranges often determine escalation of lipid-lowering therapy [1,2]. In the LDL-C interval of 4.2–4.8 mmol/L, misclassification was common for all formulas but was most pronounced for Friedewald, potentially leading to systematic underestimation of cardiovascular risk. Even modest absolute differences in LDL-C can result in different therapeutic decisions, particularly in patients near treatment thresholds.

Consistent with our findings, Capece et al. recently demonstrated that formula choice had a substantial impact on LDL-C goal achievement in hospitalized diabetic patients, underscoring the clinical relevance of identifying the most accurate estimation equation for each laboratory and population setting [12].

In a dataset of 29,504 participants from Turkey [25], the Spasić–Kotur–Vujović equation showed the best concordance with D-LDL-C when compared with traditional formulas.

Similar findings have been reported in large cohorts from India [18], Italy [26], Korea [27], and Peru [19], where the Spasić–Kotur–Vujović formula demonstrated superior agreement with D-LDL-C across a broad range of TG concentrations [27,28], and it has been explicitly recommended over the Friedewald formula in the Indian population [28,29]. Our findings add to this growing evidence base and support consideration of this formula as a preferable alternative to the Friedewald equation in routine clinical practice.

### 4.6. Strengths and Limitations

This study has several strengths. It includes a large sample size, comprehensive stratification across lipid parameters of clinical relevance, and simultaneous evaluation of multiple LDL-C estimation formulas using both analytical (bias and agreement) and clinical (classification accuracy) performance metrics. The use of real-world patient data enhances the applicability of the findings to routine laboratory and clinical practice.

Several limitations warrant consideration. The study was conducted at a single center and used a single homogeneous LDL-C assay, which may limit generalizability to other analytical platforms or populations. Beta-quantification was not performed; therefore, biological LDL-C accuracy cannot be assessed. Homogeneous LDL-C assays themselves are subject to TG- and matrix-dependent bias and are not interchangeable across manufacturers [3,5,19,24,30].

Consequently, the observed superiority of the Spasić–Kotur–Vujović formula should be interpreted as improved agreement with the specific analytical system and lipid distribution of this population. In routine practice, clinicians base decisions on LDL-C values reported by their laboratory rather than on ultracentrifugation-derived reference methods. A formula that better aligns with those reported values and reduces misclassification is therefore clinically meaningful within that context.

Inclusion of other equations (i.e., the Sampson, De Cordova, Chen equations) in future studies may provide additional insight into the performance of contemporary LDL-C estimation methods [20,31].

## 5. Conclusions

In this routine clinical laboratory setting, all four evaluated LDL-C estimation formulas systematically underestimated D-LDL-C, and the magnitude of underestimation increased at higher LDL-C, TG, and non-HDL-C concentrations. These patterns were consistent across analytical agreement metrics and treatment-relevant LDL-C categories.

Among the evaluated formulas, the Spasić–Kotur–Vujović formula showed the smallest mean bias, the narrowest limits of agreement, and the highest classification accuracy relative to the homogeneous direct LDL-C assay used in this study population. In contrast, the Friedewald formula demonstrated the largest deviations and the lowest classification accuracy, particularly in strata with higher TG and non-HDL-C levels.

These results show that analytical agreement and classification performance of LDL-C estimation formulas are strongly influenced by TG concentration and lipid profile strata. The data indicate that formula-derived LDL-C values differ systematically from D-LDL-C in this laboratory setting, particularly at higher LDL-C and triglyceride levels that are clinically decision-relevant.

All four formulas showed strong linear association with D-LDL-C but exhibited clinically relevant bias and had a lower correct classification rate in higher lipid categories. Thus, the search for the “ideal” formula for LDL-C estimation is still ongoing. It is important to emphasize that the observed superiority of the Spasić–Kotur–Vujović formula reflects its agreement with the homogeneous direct LDL-C assay performed on the Alinity c analyzer (Abbott Laboratories, Chicago, IL, USA) in this study population. Results may differ when other direct LDL-C assay platforms are used as the comparator.

## Figures and Tables

**Table 1:** Comparison of directly measured LDL-C (D-LDL-C) concentrations and LDL-C values calculated using the Spasić–Kotur–Vujović (SKV-LDL-C), Friedewald (F-LDL-C), De Long (DL-LDL-C), and Martin–Hopkins (MH-LDL-C) formulas, stratified by levels of D-LDL-C, total cholesterol (TC), triglycerides (TG), and non-HDL cholesterol (non-HDL-C), as well as in the overall study population (*n* = 3935). The table reports the mean ± standard deviation (SD) of D-LDL-C and calculated LDL-C concentrations compared by Student’s *t*-test for paired samples. Pearson’s correlation coefficients (r) were calculated to assess the linear association between directly measured and calculated LDL-C values. LDL-C concentrations are expressed in mmol/L. * *p* < 0.001, † *p* < 0.01.

**D-LDL-C, mmol/L**	***n***	**D-LDL-C**	**SKV-LDL-C**	**r**	**F-LDL-C**	**r**	**DL-LDL-C**	**r**	**MH-LDL-C**	**r**
**≤2.5**	1070	1.92 ± 0.44	1.94 ± 0.47 †	0.777	1.78 ± 0.47 *	0.771	1.90 ± 0.47 †	0.779	1.86 ± 0.46 *	0.763
**2.6–3.3**	1069	2.96 ± 0.23	2.83 ± 0.33 *	0.593	2.66 ± 0.33 *	0.577	2.78 ± 0.33 *	0.593	2.71 ± 0.32 *	0.584
**3.4–4.1**	919	3.73 ± 0.23	3.52 ± 0.36 *	0.585	3.33 ± 0.35 *	0.578	3.47 ± 0.36 *	0.587	3.38 ± 0.35 *	0.584
**4.2–4.8**	526	4.45 ± 0.20	4.13 ± 0.33 *	0.496	3.93 ± 0.34 *	0.470	4.07 ± 0.33 *	0.492	3.96 ± 0.31 *	0.498
**≥4.9**	351	5.56 ± 0.70	5.14 ± 0.74 *	0.799	4.92 ± 0.72 *	0.800	5.08 ± 0.73 *	0.801	4.94 ± 0.72 *	0.801
**Overall dataset**	3935	3.29 ± 1.16	3.13 ± 1.06 *	0.958	2.95 ± 1.04 *	0.956	3.08 ± 1.05 *	0.958	3.00 ± 1.02 *	0.957
**TC, mmol/L**	***n***	**D-LDL-C**	**SKV-LDL-C**	**r**	**F-LDL-C**	**r**	**DL-LDL-C**	**r**	**MH-LDL-C**	**r**
**≤4.1**	1240	2.11 ± 0.58	2.03 ± 0.48 *	0.847	1.88 ± 0.49 *	0.839	1.99 ± 0.48 *	0.848	1.94 ± 0.47 *	0.837
**4.2–5.1**	1233	3.17 ± 0.50	2.99 ± 0.38 *	0.802	2.82 ± 0.40 *	0.785	2.94 ± 0.38 *	0.801	2.86 ± 0.36 *	0.793
**5.2–6.1**	926	3.99 ± 0.51	3.77 ± 0.39 *	0.783	3.58 ± 0.41 *	0.775	3.72 ± 0.39 *	0.785	3.63 ± 0.37 *	0.772
**6.2–7.1**	400	4.78 ± 0.61	4.57 ± 0.44 *	0.669	4.35 ± 0.48 *	0.684	4.51 ± 0.45 *	0.677	4.40 ± 0.41 *	0.671
**≥7.2**	136	6.03 ± 0.85	5.73 ± 0.70 *	0.848	5.47 ± 0.70 *	0.868	5.66 ± 0.70 *	0.853	5.51 ± 0.69 *	0.847
**TG, mmol/L**	***n***	**D-LDL-C**	**SKV-LDL-C**	**r**	**F-LDL-C**	**r**	**DL-LDL-C**	**r**	**MH-LDL-C**	**r**
**≤1.1**	1646	3.01 ± 1.04	2.85 ± 0.93 *	0.960	2.75 ± 0.92 *	0.960	2.82 ± 0.93 *	0.960	2.70 ± 0.91 *	0.960
**1.2–1.6**	1084	3.38 ± 1.10	3.20 ± 1.0 *	0.961	3.04 ± 1.00 *	0.961	3.16 ± 1.0 *	0.961	3.07 ± 0.97 *	0.961
**1.7–2.2**	618	3.56 ± 1.26	3.37 ± 1.13 *	0.963	3.14 ± 1.13 *	0.964	3.31 ± 1.13 *	0.964	3.26 ± 1.06 *	0.963
**2.3–2.8**	309	3.61 ± 1.28	3.48 ± 1.21 *	0.956	3.17 ± 1.21 *	0.956	3.40 ± 1.21 *	0.956	3.40 ± 1.13 *	0.956
**2.9–4.4**	278	3.63 ± 1.29	3.53 ± 1.22 †	0.918	3.12 ± 1.23 *	0.917	3.42 ± 1.22 *	0.918	3.50 ± 1.10 *	0.917
**Non-HDL-C, mmol/L**	***n***	**D-LDL-C**	**SKV-LDL-C**	**r**	**F-LDL-C**	**r**	**DL-LDL-C**	**r**	**MH-LDL-C**	**r**
**≤2.5**	693	1.78 ± 0.47	1.72 ± 0.36 *	0.699	1.59 ± 0.39 *	0.688	1.68 ± 0.37 *	0.698	1.62 ± 0.35 *	0.692
**2.6–3.2**	880	2.63 ± 0.42	2.50 ± 0.28 *	0.756	2.35 ± 0.34 *	0.754	2.46 ± 0.30 *	0.757	2.39 ± 0.24 *	0.733
**3.3–3.8**	822	3.24 ± 0.41	3.06 ± 0.28 *	0.711	2.89 ± 0.35 *	0.700	3.01 ± 0.30 *	0.708	2.94 ± 0.23 *	0.706
**3.9–4.5**	735	3.87 ± 0.43	3.65 ± 0.30 *	0.711	3.45 ± 0.37 *	0.706	3.59 ± 0.31 *	0.711	3.51 ± 0.25 *	0.701
**≥4.6**	805	4.83 ± 0.73	4.62 ± 0.70 *	0.862	4.38 ± 0.73 *	0.869	4.55 ± 0.71 *	0.866	4.45 ± 0.66 *	0.860

**Table 2:** Mean absolute bias, limits of agreement, and mean percentage differences between directly measured LDL-C (D-LDL-C) and LDL-C values calculated using the Spasić–Kotur–Vujović (SKV-LDL-C), Friedewald (F-LDL-C), De Long (DL-LDL-C), and Martin–Hopkins (MH-LDL-C) formulas across stratified levels of D-LDL-C, total cholesterol (TC), triglycerides (TG), and non-HDL cholesterol (non-HDL-C). The table reports the mean absolute bias (calculated LDL-C − D-LDL-C) with corresponding limits of agreement, expressed as (Bias − 1.96 SD) to (Bias + 1.96 SD), and the mean percentage difference for each formula within the respective lipid strata. LDL-C concentrations are reported in mmol/L.

	Mean Absolute Bias [(Bias − 1.96 SD) − (Bias + 1.96 SD)]				Mean Percentage Difference (%)			
**D-LDL-C, mmol/L**	**ΔSKV-LDL-C**	**ΔF-LDL-C**	**ΔDL-LDL-C**	**ΔMH-LDL-C**	**ΔSKV-LDL-C**	**ΔF-LDL-C**	**ΔDL-LDL-C**	**ΔMH-LDL-C**
**≤2.5**	−0.02 [(−0.62)–0.58]	0.14 [(−0.47)–0.75]	0.02 [(−0.57)–0.62]	0.07 [(−0.54)–0.68]	−3.09	5.92	−0.62	1.49
**2.6–3.3**	0.14 [(−0.39)–0.66]	0.31 [(−0.23)–0.85]	0.18 [(−0.34)–0.71]	0.25 [(−0.27)–0.77]	4.60	10.34	6.17	8.51
**3.4–4.1**	0.21 [(−0.37)–0.79]	0.40 [(−0.17)–0.96]	0.26 [(−0.31)–0.83]	0.35 [(−0.22)–0.91]	5.62	10.61	6.99	9.37
**4.2–4.8**	0.33 [(−0.24)–0.89]	0.53 [(−0.06)–1.12]	0.38 [(−0.18)–0.95]	0.49 [(−0.05)–1.03]	7.36	11.85	8.58	10.98
**≥4.9**	0.40 [(−0.34)–1.13]	0.62 [(−0.11)–1.35]	0.46 [(−0.27)–1.19]	0.60 [(−0.12)–1.32]	7.07	11.06	8.16	10.75
**Overall**	0.16 [(−0.48)–0.80]	0.34 [(−0.32)–1.00]	0.21 [(−0.43)–0.85]	0.29 [(−0.38)–0.95]	3.34	9.47	5.01	7.33
**TC, mmol/L**	**ΔSKV-LDL-C**	**ΔF-LDL-C**	**ΔDL-LDL-C**	**ΔMH-LDL-C**	**ΔSKV-LDL-C**	**ΔF-LDL-C**	**ΔDL-LDL-C**	**ΔMH-LDL-C**
**≤4.1**	0.07 [(−0.46)–0.61]	0.22 [(−0.33)–0.78]	0.11 [(−0.42)–0.65]	0.17 [(−0.39)–0.72]	1.08	9.15	3.29	5.66
**4.2–5.1**	0.18 [(−0.40)–0.77]	0.35 [(−0.25)–0.96]	0.23 [(−0.36)–0.81]	0.30 [(−0.30)–0.90]	4.54	10.11	6.06	8.38
**5.2–6.1**	0.21 [(−0.41)–0.84]	0.41 [(−0.23)–1.04]	0.27 [(−0.36)–0.89]	0.35 [(−0.29)–0.99]	4.64	9.75	6.04	8.21
**6.2–7.1**	0.20 [(−0.69)–1.10]	0.42 [(−0.46)–1.30]	0.26 [(−0.62)–1.15]	0.38 [(−0.51)–1.27]	3.24	8.02	4.54	6.99
**≥7.2**	0.29 [(−0.59)–1.18]	0.56 [(−0.30)–1.42]	0.37 [(−0.51)–1.24]	0.52 [(−0.37)–1.40]	4.35	8.91	5.60	8.11
**TG, mmol/L**	**ΔSKV-LDL-C**	**ΔF-LDL-C**	**ΔDL-LDL-C**	**ΔMH-LDL-C**	**ΔSKV-LDL-C**	**ΔF-LDL-C**	**ΔDL-LDL-C**	**ΔMH-LDL-C**
**≤1.1**	0.16 [(−0.38)–0.69]	0.26 [(−0.29)–0.80]	0.18 [(−0.35)–0.72]	0.30 [(−0.24)–0.85]	3.73	7.57	4.78	9.18
**1.2–1.6**	0.18 [(−0.43)–0.78]	0.34 [(−0.26)–0.95]	0.22 [(−0.39)–0.83]	0.31 [(−0.31)–0.94]	4.03	9.68	5.58	8.26
**1.7–2.2**	0.19 [(−0.50)–0.88]	0.42 [(−0.26)–1.11]	0.25 [(−0.43)–0.94]	0.31 [(−0.42)–1.03]	3.11	10.86	5.23	6.14
**2.3–2.8**	0.13 [(−0.60)–0.87]	0.44 [(−0.29)–1.17]	0.22 [(−0.53)–0.96]	0.21 [(−0.54)–0.97]	2.13	12.17	4.87	3.58
**2.9–4.4**	0.10 [(−0.91)–1.10]	0.51 [(−0.50)–1.53]	0.21 [(−0.80)–1.22]	0.13 [(−0.89)–1.15]	0.15	13.78	3.88	−0.41
**Non-HDL-C, mmol/L**	**ΔSKV-LDL-C**	**ΔF-LDL-C**	**ΔDL-LDL-C**	**ΔMH-LDL-C**	**ΔSKV-LDL-C**	**ΔF-LDL-C**	**ΔDL-LDL-C**	**ΔMH-LDL-C**
**≤2.5**	0.05 [(−0.50)–0.59]	0.18 [(−0.39)–0.75]	0.08 [(−0.47)–0.64]	0.14 [(−0.41)–0.69]	−0.13	8.19	2.15	5.38
**2.6–3.2**	0.13 [(−0.47)–0.67]	0.28 [(−0.41)–0.67]	0.17 [(−0.36)–0.71]	0.24 [(−0.33)–0.81]	3.54	9.78	5.24	7.58
**3.3–3.8**	0.18 [(−0.39)–0.74]	0.35 [(−0.24)–0.94]	0.23 [(−0.34)–0.79]	0.30 [(−0.28)–0.88]	4.31	10.04	5.88	7.99
**3.9–4.5**	0.22 [(−0.37)–0.82]	0.42 [(−0.19)–1.03]	0.28 [(−0.32)–0.87]	0.36 [(−0.25)–0.97]	5.16	10.39	6.59	8.57
**≥4.6**	0.21 [(−0.64)–1.06]	0.46 [(−0.37)–1.29]	0.28 [(−0.56)–1.12]	0.38 [(−0.48)–1.25]	3.44	8.80	4.91	6.93

**Table 3:** Classification accuracy of the Spasić–Kotur–Vujović (SKV-LDL-C), Friedewald (F-LDL-C), De Long (DL-LDL-C), and Martin–Hopkins (MH-LDL-C) formulas across stratified levels of directly measured LDL-C (D-LDL-C), total cholesterol (TC), triglycerides (TG), non-HDL cholesterol (non-HDL-C). The table presents the percentage of participants classified by each formula within the corresponding lipid strata relative to D-LDL-C concentrations. Classification performance was evaluated across clinically relevant lipid categories to assess the agreement between calculated and D-LDL-C values. U—underestimated; P—properly classified; O—overestimated.

**D-LDL-C, mmol/L**	***n***	**SKV-LDL-C**			**F-LDL-C**			**DL-LDL-C**			**MH-LDL-C**		
		**U**	**P**	**O**	**U**	**P**	**O**	**U**	**P**	**O**	**U**	**P**	**O**
**≤2.5**	1070	/	96	4	/	98	2	/	96	4	/	97	3
**2.6–3.3**	1069	22	74	3	41	58	1	27	70	3	35	62	3
**3.4–4.1**	919	38	59	3	58	41	1	43	55	2	54	44	2
**4.2–4.8**	526	60	39	1	80	20	0	65	35	0	77	23	0
**≥4.9**	351	39	61	0	53	47	0	45	55	0	52	48	0
**Overall**	3935	27	70	3	40	58	2	29	68	3	37	61	2
**TC, mmol/L**	***n***	**SKV-LDL-C**			**F-LDL-C**			**DL-LDL-C**			**MH-LDL-C**		
**≤4.1**	1240	13	86	1	18	81	1	15	84	1	18	81	1
**4.2–5.1**	1233	30	67	3	48	51	1	32	65	3	42	56	2
**5.2–6.1**	926	38	57	5	56	42	2	41	55	4	52	45	3
**6.2–7.1**	400	35	57	8	56	41	3	42	53	5	53	43	4
**≥7.2**	136	4	95	1	13	86	1	6	93	1	9	90	1
**TG, mmol/L**	***n***	**SKV-LDL-C**			**F-LDL-C**			**DL-LDL-C**			**MH-LDL-C**		
**≤1.1**	1646	24	75	1	33	66	1	26	72	2	37	63	1
**1.2–1.6**	1084	28	69	3	41	58	1	32	66	2	39	60	1
**1.7–2.2**	618	29	69	2	48	51	1	34	64	2	37	62	1
**2.3–2.8**	309	26	68	6	50	48	2	33	62	5	34	61	5
**2.9–4.4**	278	29	64	7	52	45	3	34	60	6	30	60	10
**Non-HDL-C, mmol/L**	***n***	**SKV-LDL-C**			**F-LDL-C**			**DL-LDL-C**			**MH-LDL-C**		
**≤2.5**	693	3	97	0	3	97	0	3	97	0	3	97	0
**2.6–3.2**	880	26	71	3	38	60	2	30	68	2	41	59	0
**3.3–3.8**	822	34	63	3	52	47	1	39	59	2	44	54	2
**3.9–4.5**	735	36	60	4	53	45	2	41	56	3	47	50	3
**≥4.6**	805	31	62	7	53	45	2	37	59	4	47	48	5

## Data Availability

The data presented in this study are available upon reasonable request from the first author.
